# Derivation of Particle Number Concentration from the Size Distribution: Theory and Applications

**DOI:** 10.1021/acs.analchem.4c05990

**Published:** 2025-05-16

**Authors:** Natalia Farkas, John A. Kramar, Antonio R. Montoro Bustos, George Caceres, Monique Johnson, Matthias Roesslein, Elijah J. Petersen

**Affiliations:** † Microsystems and Nanotechnology Division, Physical Measurement Laboratory, NIST, Gaithersburg, Maryland 20899, United States; ‡ United States Department of Agriculture, 110295 Forest Products Laboratory, Madison, Wisconsin 53726, United States; § Chemical Sciences Division, Material Measurement Laboratory, NIST, Gaithersburg, Maryland 20899, United States; ∥ Particles-Biology Interactions Laboratory, Empa, Swiss Federal Laboratories for Material Testing and Research, St. Gallen 9015, Switzerland; ⊥ Biosystems and Biomaterials Division, Material Measurement Laboratory, 10833 National Institute of Standards and Technology (NIST), Gaithersburg, Maryland 20899, United States

## Abstract

The particle number concentration (PNC) in a suspension is a key measurand in nanotechnology. A common approach for deriving PNC is to divide the total mass concentration by the per-particle mass, calculated as density times volume. The volume is most frequently derived from the arithmetic mean diameter (AMD) of the size distribution. The harmonic mean volume (HMV) has also been used. Given a known size distribution, we show that the correct PNC is obtained by using the arithmetic mean volume (AMV). The AMD-based volume results in an overestimate in PNC that increases superlinearly with increasing coefficient of variation (CV), reaching 12% at CV = 0.2 for a normal distribution. HMV would yield a much greater overestimate, exceeding 50%. The error in the AMD-derived PNC shows only weak skew dependence, suggesting a simple approximate correction as a function of CV in the common situation where AMD and CV are known but the overall size distribution is unknown. Using published data sets of gold nanoparticles, we demonstrate an overall consistency of ±1.1% in comparing the PNC directly determined by single-particle inductively coupled plasma–mass spectrometry (spICP-MS) and the PNCs derived from AMV using size distributions independently measured by high-resolution scanning electron microscopy and spICP-MS. We further compare AMV and AMD-derived PNCs for well-characterized polystyrene nanoparticle standards, illustrating sensitivity to distributional characteristics along with common errors to avoid. Nanoparticles in environmental samples, food additives, and nanomedicines often have CVs greater than 0.3, for which uncorrected AMD-derived PNC errors can exceed 35%.

## Graphical abstract

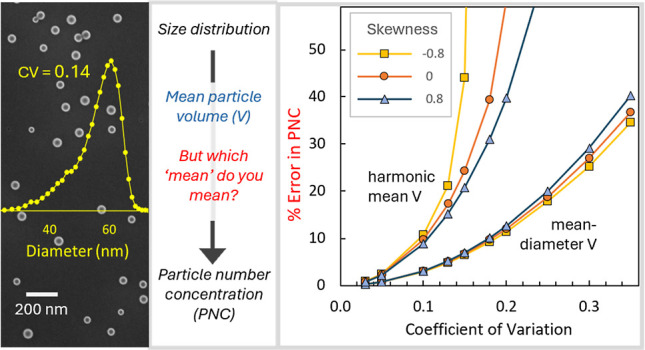

## Introduction

The particle number concentration (PNC) of a nanoparticle suspension is a key measurand in many nanotechnology applications.^1–3^ For example, it can be a critical attribute for nanomedicine dosing^4,5^ and can be valuable for assessing the stability of a nanomedicine formulation across time and for determining the intended dilution prior to administration.^6^ Likewise, determining the PNC of released particles from different industrial processes, such as chemical mechanical polishing of silicon dioxide using ceria slurry and fixed abrasives, is highly important.^7^ The measurement of the PNC also has regulatory importance,^8^ such as the proposed European Union definition^9^ for labeling of products containing nanomaterials (50% of the particles between 1 and 100 nm). In addition, the PNC is a key measurand for assessing the potential ecological and human health toxicological effects of suspended nanoparticles.^10,11^ Some studies show that PNC is better correlated with chemical activity than the more commonly used total mass concentration.^12^

Despite its conceptual simplicity and widely recognized basic importance in characterizing particle suspensions, PNC remains a challenging measurand to determine accurately.^13–16^ For metallic and stable oxide nanoparticles, direct determination of PNC at environmentally relevant concentrations (on the order of 1 ng L^–1^) is possible with single-particle inductively coupled plasma−mass spectrometry (spICP-MS).^17,18^ However, to have superior accuracy and confidence in the measurement, even for this particle-by-particle detection method, a nanoparticle reference standard with a known concentration is needed for accurately determining the transport efficiency, which is defined as the fraction of introduced sample that is transported to the plasma.^19,20^ Some efforts have been made to directly count the number of particles microscopically, e.g., by depositing a small, known-volume droplet of a low concentration suspension onto a substrate and individually counting the number of particles after liquid evaporation.^21^ A more conceptually straightforward approach is to simply count all the particles within the focal volume of an optical microscope at a suitable dilution, but this method is susceptible to uncertainty in determining the illuminated and in-focus volume and to the challenge of detecting and accurately counting the smaller particles in the distribution, especially if fluorescent markers are not an option. Validated techniques that span the full range of materials, types, shapes, and sizes of particles are not available,^8^ and there is a shortage of nanoparticle reference materials with certified PNC values for the evaluation of accuracy.^22–24^

In addition to direct particle counting methods, perhaps the most widely used method of determining PNC for nanoparticles composed of a uniform material is to measure the total mass concentration (TMC), *C,* of the material in the suspension, combined with knowledge of the particle volume *V* and density ρ.^25^ For a suspension of particles of uniform size, PNC is given simply by *PNC* = *C*/(ρ *V*). The challenge we address here is how to more accurately handle a suspension that consists of a distribution of particle sizes.

In this paper, we present the determination of the PNC of a particle suspension from the TMC by using the arithmetic mean volume (AMV) of the particle size distribution. We compare this to the use of the arithmetic mean diameter (AMD)-based volume, as often found in the literature, or the use of the harmonic mean volume (HMV).^6,17,18,26–30^ We investigate the magnitude of the error that results if the mean volume is thus incorrectly calculated from the particle size distribution, including the dependence of the error on the width and skew of the distribution. Based on the theoretical analysis, we propose a simple coefficient of variation (CV)-dependent PNC error correction that can be applied to the AMD-derived PNC in circumstances where only the mean size and CV of the distribution are known. We demonstrate the use of this correction by applying it to a well-characterized polystyrene nanoparticle standard having a less-than-ideal distribution. We show consistency of the calculation through determination of the PNCs of gold nanoparticle suspensions of different sizes and surface coatings using the AMVs from independent, high-resolution scanning electron microscope (HR-SEM)^31^ and spICP-MS particle size distribution measurements and comparisons of these results with direct determination of PNC by spICP-MS.^18^ We also show the beneficial application of the correction in a real-world food additive application.

## Background

### PNC Derivation

To calculate PNC from TMC, we must determine how many particles are needed to sum to the total particle mass in a given suspension volume. Under the assumption that the particle density is known, the mass for each individual particle is the product of the particle volume *V* and density ρ. For the total number *N* of particles in the suspension volume, the total mass is simply the density times the sum of the volumes of each individual particle, ρ Σ*V*_*i*_. Conversion to PNC from TMC, *C*, for a suspension of particles is then

1 $${PNC}_{AMV}=C\frac{N}{\rho \sum V_{i}}=\frac{C}{\rho {\mathrm{V̅}}_{A}}$$

Where the conversion factor $\frac{N}{\rho \sum V_{i}}$ is simply the inverse of the product of the density and the AMV of all the particles in the ensemble, ${\mathrm{V̅}}_{A}=\frac{1}{N}\sum V_{i}$ . If *s*_*i*_ is the spherical-equivalent-volume diameter of the *i*th particle, then ${\mathrm{V̅}}_{A}=\frac{\pi }{6}\frac{1}{N}\sum {s_{i}}^{3}$ . This ${\mathrm{V̅}}_{A}$ for a discrete set of particles is equivalent to the arithmetic mean volume derived from a probability density function (PDF) description of the particle size distribution.

### Current Practice

While this conversion of TMC to PNC is straightforward, unfortunately there are many examples in the literature where two different approaches have been taken that are not mathematically equivalent to the AMV-based PNC derivation. The first, more common approach, is to calculate the mean volume for the TMC-to-PNC conversion from the AMD of the particles $\mathrm{s̅}=\frac{1}{N}\sum s_{i}$ .^6,27,29,30^ This approach results in a mean-diameter volume $V_{\mathrm{s̅}}=\frac{\pi }{6}{\left(\frac{1}{N}\sum s_{i}\right)}^{3}$ . Irrespective of the width, skew, or multimodality of the underlying size distribution, $V_{\mathrm{s̅}}$ is always smaller than ${\mathrm{V̅}}_{A}$ (except in the limiting case where all the particles are the same size), leading to an overestimate of the PNC. While it is not accurate to use $V_{\mathrm{s̅}}$ as the mean volume for converting TMC to PNC, the approximation is nevertheless of some use and is frequently unavoidable if reliable information about the particle size distribution is not available. We use *PNC_AMD_* to denote the PNC calculated using $V_{\mathrm{s̅}}$ . Putting it in the form of eq 1

2 $${PNC}_{AMD}=\frac{C}{\rho V_{\mathrm{s̅}}}$$

In the second incorrect approach, the error in the TMC-to-PNC conversion seems to arise from starting with the PNC equation for each particle size and attempting to take the mean of these individual-size PNCs, weighted by the size distribution.^18,26,28^ This can be done within the PDF formalism, but in the discrete form, it is

3 $${PNC}_{HMV}=\frac{1}{N}\sum {PNC}_{i}$$

$${PNC}_{HMV}=\frac{1}{N}\sum \frac{C}{\rho V_{i}}=\frac{C}{\rho }\frac{1}{N}\sum \frac{1}{V_{i}}$$

Again, putting it into the same form as eq 1 yields

4 $${PNC}_{HMV}=\frac{C}{\rho {\mathrm{V̅}}_{H}}$$

where ${\mathrm{V̅}}_{H}={\left[\frac{1}{N}\sum \frac{1}{V_{i}}\right]}^{-1}$ . The mean volume ${\mathrm{V̅}}_{H}$ that we have arrived at in this case is the mathematical definition of the HMV of the particle size distribution. Similar to $V_{\mathrm{s̅}}$ , ${\mathrm{V̅}}_{H}$ is always smaller than ${\mathrm{V̅}}_{A}$ , only more so. We note in passing that if the mean of the individual *PNC*_*i*_ in eq 3 had been calculated as a harmonic mean instead of as an arithmetic mean—as is appropriate when the physical quantity *x* for which you are calculating the mean appears in the formula as 1/*x*—then eq 3 would have been

$${PNC}_{HMC}={\left[\frac{1}{N}\sum \frac{1}{{PNC}_{i}}\right]}^{-1}$$

where the subscript HMC refers to the harmonic mean of the PNCs corresponding to each particle size, and upon substitution and rearrangement, the result would have been identical to eq 1.

## Results and Discussion

### Theoretical Comparison of the Different PNC Derivations

To illustrate the effect on the derived PNC of using the different mean-volume definitions described above, the derivation formulas were applied to model PDF curves that mimic the characteristics of actual particle size distributions. Commonly observed particle suspensions have size distributions of varying widths, sometimes nearly symmetric but often skewed to the left or right, sometimes significantly. Distributions that skew to the right are often found to approximate log-normal distributions, having positive skewness, where skewness is defined as the standardized third moment of the distribution, γ_1_. We generated model PDF curves using the skew normal distribution function with skew parameters of −3, 0, and 3 and with the location and scale parameters adjusted such that the means of all the generated particle size distributions are 1 and the CVs range from 0.03 to 0.35; the CV is the ratio of the standard deviation, σ, relative to the mean, μ. We chose the skew normal distribution because it conveniently produces mirror-image distributions having positive and negative skewness. We found no difference in the derived PNC between using the skew normal and log-normal distributions if the generating parameters are adjusted such that the distributions have the same mean, CV, and skewness, even though the shapes of the PDFs are not identical. Skew normal distributions with skew parameters of ±3 have a skewness of approximately ±0.8. A subset of the PDF curves we used is shown in Figure 1a. Truncation and minor scaling adjustments were necessary for the negative-skewness PDF curves having CV larger than 0.18 because of the tailing of the mathematical function into negative values, which is not physically possible for particle diameters. This led to smaller skewness amplitudes in those cases, reaching a minimum skewness amplitude of −0.5 for the PDF with a CV of 0.35.

**Figure 1:**
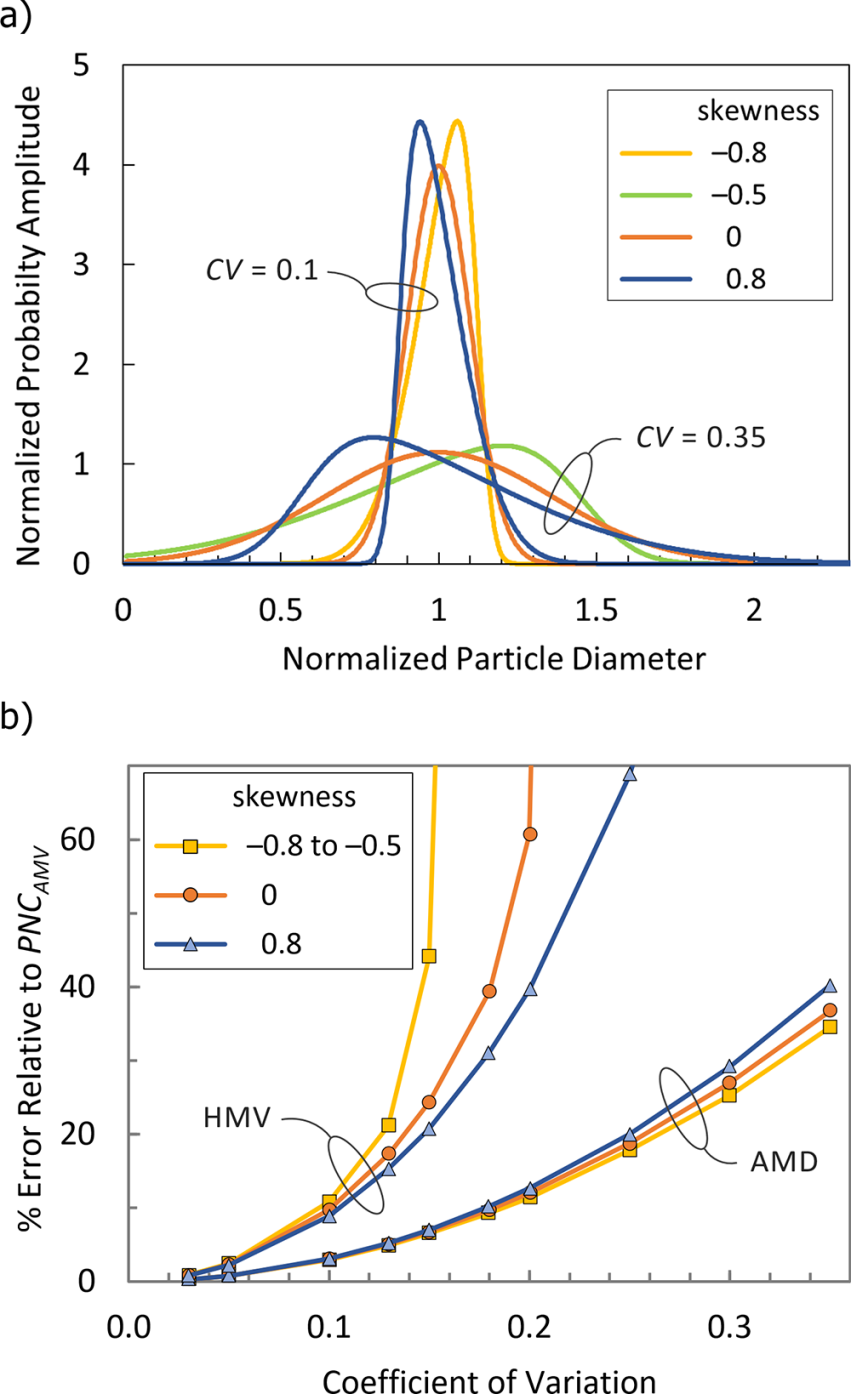
(a) Representative PDF curves used for demonstrating the effects of using different ways of calculating the mean volume of a particle size distribution for deriving PNC from TMC. The PDF curves are generated from the skew normal function with the skew parameter set to −3, 0, or 3, and the location and scale parameters adjusted such that the mean particle diameter is one and the CVs are the selected values. (b) PNC errors relative to *PNC_AMV_* as a function of the CV and skewness of the particle size distribution, based on using either the HMV or the volume derived from the AMD.

For this set of PDFs, the relative error in using either the AMD-based volume or the HMV for calculating PNC is presented in Figure 1b. We note that correctly accounting for the distributional nature of the particles by using AMV in the PNC calculation results in a smaller PNC than that calculated using the AMD-based volume. The error in *PNC_AMD_* increases superlinearly with increasing CV, reaching almost 40% as the CV reaches 0.35. For CV below 0.1, as would be typical for reference materials and most commercially available metallic particle samples, the maximum relative error in in PNC from using the AMD-based volume instead of AMV is only 3%. The skew dependence of the *PNC_AMD_* error is weak, affecting the magnitude of the error by less than 3% for the range of skews and CVs explored. On the other hand, using HMV to calculate the PNC results in a much greater error that increases precipitously as the CV increases beyond 0.1, soon exceeding 100%, and is also strongly affected by skew.

### Error Correction for the AMD-Derived PNC

It bears noting that if the full probability density function for a particle suspension were not available, as is frequently the case, and if all that one had were a reliable arithmetic mean diameter and standard deviation of the particle size distribution, one could still significantly improve the estimate of the PNC by calculating *PNC_AMD_* and correcting the result based on the known CV. For a normal distribution, the correction factor is exactly

5 $$\kappa =\frac{{PNC}_{AMV}}{{PNC}_{AMD}}={\left[1+3\;{\left(\frac{\sigma }{\mu }\right)}^{2}\right]}^{-1}$$

and is shown in Figure 2 as the zero-skewness curve. This correction factor can be approximately applied to any *PNC_AMD_*-to-*PNC_AMV_* correction based on CV alone. Ignoring any skew or the possibility of a symmetric distribution that is not Gaussian only weakly affects the accuracy of the correction, as seen in Figure 2 for skew parameters of ±3. The derivation of the correction factor in eq 5 and the recognition of its approximate generality are a key practical contribution of this work, given that the full details of the size distributions of particle suspensions are often not known.

**Figure 2:**
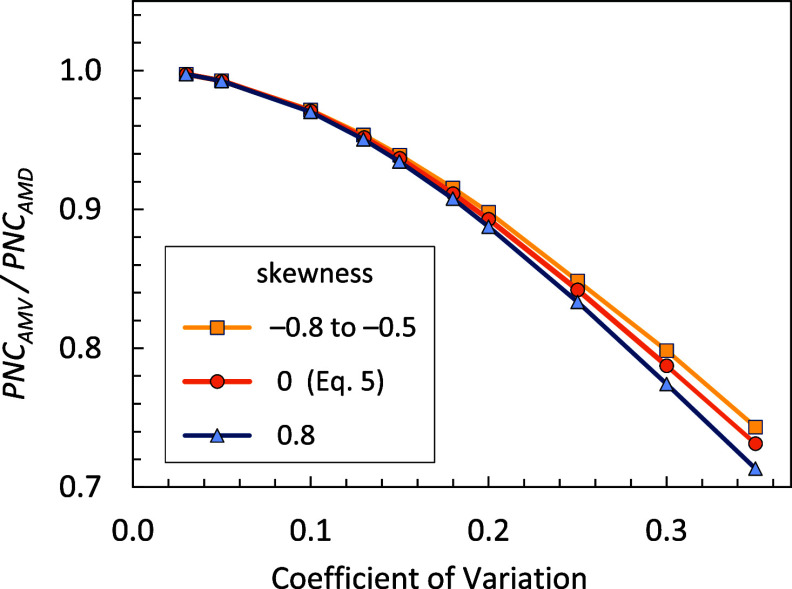
Correction factors for converting *PNC_AMD_* to *PNC_AMV_* for normal distributions and skew-normal distributions with skew parameters of ±3. The normal distribution (skew = 0) curve is a plot of κ from eq 5.

As an example of using eq 5 to correct *PNC_AMD_*, we apply it to the PDF of the SRM 1964 reference standard,^32^ a 60 nm nominal diameter polystyrene latex, shown in Figure 3. Numerical calculations using the given PDF data yield a mean diameter of 55.7 nm, which corresponds to a $V_{\mathrm{s̅}}$ volume of 90.5 × 10^3^ nm.^3^ This can be compared with a calculated arithmetic mean ${\mathrm{V̅}}_{A}$ volume of 95.7 × 10^3^ nm.^3^ The relative error in PNC from using the AMD-based volume instead of the AMV is +5.7%. But using eq 5 and the calculated CV of 0.142 gives a correction factor of 0.943, which when applied to the erroneous *PNC_AMD_* value reduces the PNC error to −0.3%. As a graphical illustration, the dotted line in Figure 3 represents the mean diameter, and the gray curve is a normal distribution centered at the mean diameter and having a CV of 0.142. Unintuitively, the highly asymmetric PDF of SRM 1964 having a skewness of −1.1 yields the same AMV, to within 0.3%, as a normal distribution having the same mean diameter and CV.

**Figure 3:**
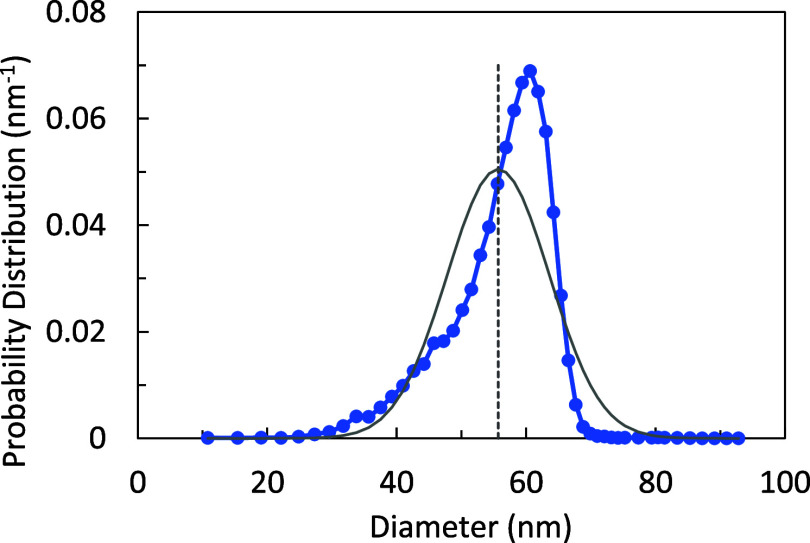
SRM 1964 size probability distribution function (blue circles). The mean diameter is indicated by the dashed line. The solid gray line is a normal distribution constructed from the numerical mean diameter and standard deviation calculated from the size distribution data. The *PNC_AMV_* calculated from these two distributions is the same to within 0.3%.

A couple of cautions are in order. In this example, the full particle size distribution is available, permitting an accurate numerical calculation of the mean diameter and CV. One should not assume such accuracy for a PNC calculated from an unknown underlying distribution for which one only has the manufacturer supplied mean diameter and a CV of unknown uncertainty. More significantly, the certified reference size value given for SRM 1964 is the modal diameter, not the mean diameter. The modal diameter is a practical measurand if the reference material is being used to calibrate magnification or scale, since the mode can be more accurately approximated than the mean when using a small number of randomly selected individual particle measurements. However, if one were to incautiously use the modal diameter of 60.4 nm instead of the mean diameter of 55.7 nm for calculating PNC via eq 2, the uncorrected result would be −18% relative to the correct *PNC_AMV_*. Furthermore, if the error correction presented in eq 5 were then applied to this incorrect modal-diameter-derived PNC value—in this case, using a CV of 0.08 based on the estimated width of the modal peak—it would result in a PNC that is smaller yet than the correct *PNC_AMV_* by an additional 2%.

In contrast to this example, SRM 1963 (a.k.a. JSR SC-010-S) is a nominal 100 nm diameter polystyrene latex reference material^33^ with one of the narrowest distributions ever reported, having a measured CV of 0.02.^34^ Using eq 5, this CV would correspond to a fractional correction of only −8.7 × 10^–4^ for converting from *PNC_AMD_* to *PNC_AMV_*. This is completely negligible relative to the 2.8% range in the reported mean diameter measurements of the material obtained using four independent traceable methods, which would result in an 8.4% range in derived PNC values.^34^ This case points to both the advantages of narrow-distribution nanoparticle suspensions for PNC and size reference standards and to the significant challenges in understanding and correcting for all measurement biases in nanoparticle size measurements more accurately than two or three percent, even among national metrology institutes using their most reliable and well-characterized methods.

### Application of the AMV-Based PNC Derivation to Gold Nanoparticle Data

To demonstrate the applicability of the *PNC_AMV_* calculation described in this work, we apply it to previously published data for selected gold nanoparticle suspensions of different sizes and surface coatings.^18,31^ We derive PNC values from the independent HR-SEM and spICP-MS size distribution measurements and compare them with direct determination of PNC by spICP-MS. HR-SEM is one of the most reliable techniques for providing a realistic particle size distribution for nearly spherical nanoparticles. When combined with an interferometer stage or calibrated with a traceable artifact, it has been validated as a reference method for ensuring material conformity and establishing traceability for nanoparticle size measurements. spICP-MS performs particle-by-particle detection on very dilute suspensions of nanoparticles (<10^8^ L^–1^) by operating in time-resolved mode with short integration times. Under such conditions, nanoparticles are identified as transient signal pulses superimposed on the steady-state background. The direct determination of PNC, i.e., *PNC_direct_*, for a nanoparticle suspension is achieved by counting the number of acquired particle events per volume of solution introduced into the ICP-MS. The signal intensity of each pulse is proportional to the mass of each individual nanoparticle, which can be translated into a size, thereby also yielding a size distribution for the nanoparticle suspension, from which the *PNC_AMV_* can be independently calculated.

Accurate calibration of the fraction of the introduced sample that is transported to the plasma, termed transport efficiency, is required for accurate spICP-MS measurement of *PNC_direct_*. In this case, the transport efficiency was determined by the particle frequency method^19^ using NIST RM 8013 as the calibration standard. Its PNC calibration value was computed via eq 2 (*PNC_AMD_*) in our previous works,^18,26^ using the knowledge of the total mass fraction, the consensus spherical-equivalent mean size of the sample, and the density of the particle.

In this work, we correct the *PNC_AMD_* calibration value for NIST RM 8013 that was used for determining the spICP-MS transport efficiency by applying the correction factor from eq 5 using the CV calculated from the particle size distribution contained in the Certificate of Analysis.^35^ The corrected PNC value is used to recalculate the *PNC_direct_* values from the previously published spICP-MS measurements.^18^ It is only a 2% correction for this relatively narrow-distribution reference material. Table 1 shows the resulting *PNC_direct_* values and the *PNC_AMV_* values derived from the corresponding spICP-MS and HR-SEM size distribution measurements.^31^

**Table 1:** Comparison of the *PNC_direct_* Values Measured by spICP-MS^18^ and the *PNC_AMV_* Values Derived from Size Distributions Measured Independently by spICP-MS and by HR-SEM,^31^ for Selected Gold Nanoparticle Suspensions of Different Sizes and Coatings^a^

	spICP-MS				HR-SEM		combined value	
	*PNC_direct_* (L^–1^)	*u* (%)	*PNC_AMV_* (L^–1^)	*u* (%)	*PNC_AMV_* (L^–1^)	*u*(%)	*PNC_avg_* (L^–1^)	*u* (%)
RM 8012 (30 nm)	2.33 × 10^14^	1.5	2.28 × 10^14^	0.9	2.35 × 10^14^	0.4	2.32 × 10^14^	1.6
30 nm PVP	2.15 × 10^14^	4.7	2.14 × 10^14^	1.4	2.36 × 10^14^	0.8	2.22 × 10^14^	5.8
30 nm bPEI	1.99 × 10^14^	9.3	1.99 × 10^14^	1.5	1.91 × 10^14^	0.9	1.96 × 10^14^	2.3
30 nm PEG	2.12 × 10^14^	6.1	1.91 × 10^14^	1.6	2.06 × 10^14^	1.1	2.03 × 10^14^	5.3
RM 8013 (60 nm)	3.07 × 10^13^	1.3	3.00 × 10^13^	1.0	3.05 × 10^13^	0.6	3.04 × 10^13^	1.1
60 nm PVP	2.63 × 10^13^	1.3	2.86 × 10^13^	1.7	2.72 × 10^13^	0.7	2.73 × 10^13^	4.3
60 nm bPEI first lot	2.41 × 10^13^	4.6	2.67 × 10^13^	1.5	2.65 × 10^13^	1.0	2.58 × 10^13^	5.6
60 nm bPEI second lot	2.74 × 10^13^	5.8	2.91 × 10^13^	1.4	3.03 × 10^13^	0.7	2.90 × 10^13^	5.0
60 nm PEG first lot	2.93 × 10^13^	1.4	2.94 × 10^13^	1.7	2.82 × 10^13^	1.5	2.90 × 10^13^	2.3
60 nm PEG second lot	2.60 × 10^13^	4.8	2.73 × 10^13^	1.5	2.55 × 10^13^	0.9	2.63 × 10^13^	3.5
100 nm citrate	5.64 × 10^12^	6.7	5.88 × 10^12^	1.7	5.40 × 10^12^	0.8	5.64 × 10^12^	4.2
100 nm PVP	4.59 × 10^12^	6.8	4.90 × 10^12^	1.6	5.01 × 10^12^	0.6	4.83 × 10^12^	4.5
100 nm bPEI	4.83 × 10^12^	4.0	4.75 × 10^12^	1.3	4.74 × 10^12^	0.8	4.77 × 10^12^	1.0
100 nm PEG	6.20 × 10^12^	1.5	5.75 × 10^12^	1.6	6.69 × 10^12^	1.3	6.21 × 10^12^	7.6

a For details, see ref 18. The combined value, *PNC_avg_*, of the three PNC measurements is also presented. Relative uncertainties, *u*, are 1σ and represent type A repeatability only.

The measurements of the three PNC values are based on different analytical and instrumental principles and are considered independent. We combine the values by averaging, *PNC_avg_*, also shown in Table 1. Figure 4 illustrates the relative deviation of *PNC_direct_* and *PNC_AMV_* derived from spICP-MS and HR-SEM size-distribution measurements with respect to *PNC_avg_*.

**Figure 4:**
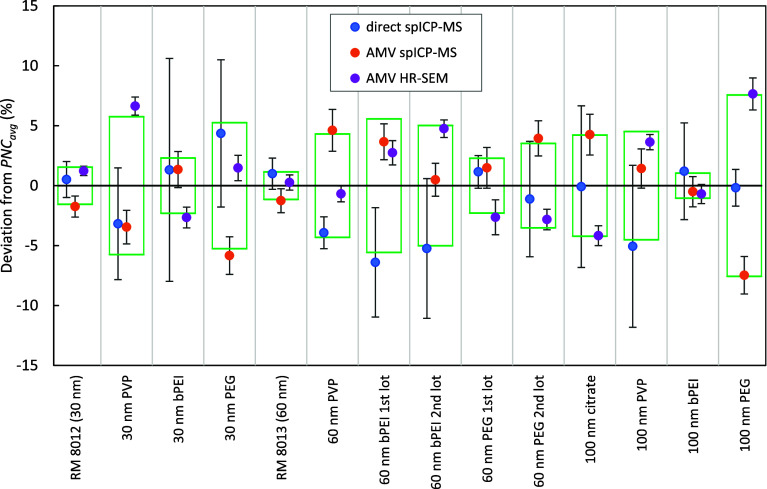
Plot of the relative deviations of *PNC_direct_* and *PNC_AMV_* derived from spICP-MS and HR-SEM size distribution measurements with respect to the combined value, *PNC_avg_*, for a set of gold nanoparticle samples. Uncertainty bars represent 1σ measurement repeatability, as in Table 1. Green boxes represent ±σ for the average of the three component values to guide the eye.

What is clear from Figure 4 is that across this set of sample measurements there is no statistically meaningful bias between these three different independent ways of determining PNC. The mean deviations from *PNC_avg_* of the three PNC determinations are only −1.1%, 0.1%, and 1.1% for *PNC_direct_*, spICP-MS *PNC_AMV_*, and HR-SEM *PNC_AMV_*, respectively; these are well within the standard deviations of each set, which are 3.1%, 3.7%, and 3.7%. It appears that there are unknown effects that are causing the PNC measurements of the individual suspensions—using any of the three methods—to deviate from the true value by more than the observed statistical variation of the individual suspension measurements. Interestingly, whatever these unknown effects are, they seem to vary randomly between particle types within this set of measurements such that none of the methods measure meaningfully higher or lower than the others on average.

In contrast to the statistical agreement among the *PNC_AMV_* and *PNC_direct_* measurements for the gold nanoparticle suspensions within the set, if we compare the *PNC_AMD_* values to *PNC_direct_*, they are on average 6.7% and 6.1% larger for the spICP-MS and HR-SEM measurements, respectively. These are statistically meaningful biases and reinforce the benefit of correctly using the AMV to calculate PNC instead of using the AMD-based volume. Moreover, a similar trend is observed when restricting to only the spICP-MS data where the use of the AMV as compared to AMD-based volume in the PNC calculations reduces the bias with respect to *PNC_direct_* from a mean value of 6.7% to 1.3%.

### Real-World Applications

All of the nanoparticle examples we have discussed so far have been polystyrene reference materials or typical commercially available metallic nanoparticles, both of which usually exhibit relatively narrow size distributions. For many product and environmental samples, the nanoparticle suspension has a much broader size distribution such that the PNC error from using the AMD-based volume instead of the AMV can be substantially greater. As an example, silicon dioxide powder is a common anticaking food additive that has attracted attention recently because of the possible presence of nanoparticles that could potentially have negative health effects.^36^ Figure 5 shows the particle size distribution of a typical SiO_2_ product received from a commercial supplier as measured by spICP-MS after suspension in water. Numerical analysis of the full distribution yields a mean diameter of 344 nm with a CV of 0.32 and a skewness of 2.0. Using the AMD-based volume instead of the AMV to calculate PNC results in a 36% error. However, the CV-based correction of eq 5 reduces the error significantly to 4.8% despite the large skew.

**Figure 5:**
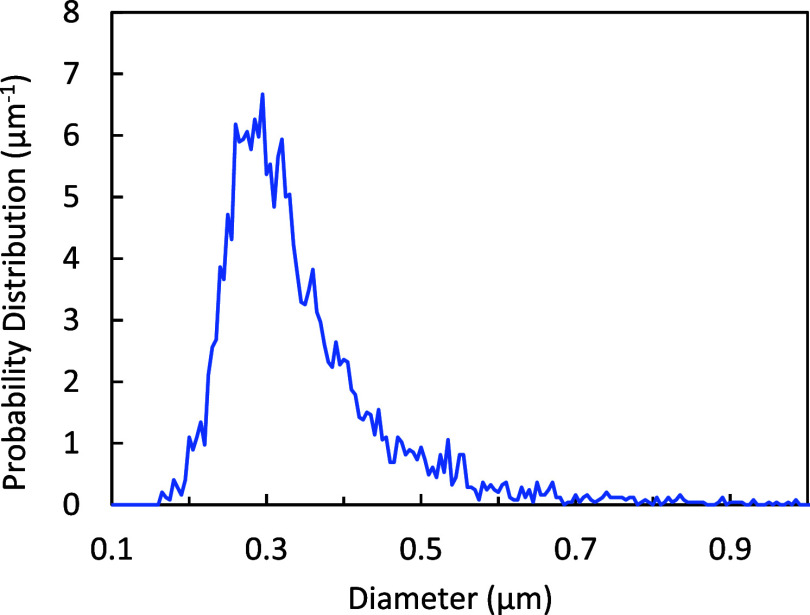
SiO_2_ particle size distribution as received from the manufacturer, measured by spICP-MS after suspension in water (data replotted from ref 36).

Our focus in this work is only on how the choice of size-distribution averaging method affects the derivation of PNC from a known mass concentration, but it is worth acknowledging that there are a host of issues that can affect the size-distribution measurement itself.^37^ The SiO_2_ example nicely illustrates two that are prevalent and significant. The first has to do with the tendency of particle samples to agglomerate, whether in suspension or during sample preparation prior to analysis. Electron microscopy images included in the referenced work show substantial agglomeration corresponding to the long tail of larges seen in Figure 5, since spICP-MS regards each of these agglomerates as a single entity. Using other counting rules as proposed in ref 38, including counting each primary particle within the agglomerates, would yield a vastly different size distribution and PNC result. This highlights the critical impact of the measurand definition on the measurement results. Another issue illustrated by this example is the size-sensitivity difference between measurement techniques. The electron microscopy images show that the primary particles comprising the agglomerates have a diameter of about 30 nm. Isolated primary particles are rare even after sonication and filtering; nevertheless, it appears that there may be a significant population of agglomerates that are smaller than 100 nm, which could be of key interest with respect to the EU definition of a nanomaterial.^9^ These smaller-size agglomerates are below the SiO_2_ detection-size threshold for spICP-MS. They are also invisible to the dynamic light scattering (DLS) measurements since their scattered-light signal is overwhelmed by the larger agglomerates. Clearly, if these smaller particles were measured and included in the size distribution, they could have a marked effect on the inferred PNC. Unfortunately, many nanoparticle studies in the literature do not address these size-distribution measurement challenges. A frequent default is to rely on distributional representations derived from DLS measurements alone, which are prone to large errors due to a variety of issues as we have previously shown in detail.^40^

## Conclusions

Historically in the literature, different mean-volume calculation methods have frequently been used for deriving the PNC from the total mass concentration without rigorous justification, the AMD-based volume approach being the most common. We have shown instead that the arithmetic mean of the individual particle volumes, AMV, is the correct way to account for the fact that particle samples always have a distribution of sizes. This is significant because the error in using *PNC_AMD_* instead of *PNC_AMV_* can be quite large for broader distributions, reaching nearly 20% for a normal distribution with a CV of 0.25 and more than 35% for a commercial product example having a highly skewed distribution and a CV of 0.32.

To demonstrate the application of the AMV-based PNC, we compared *PNC_AMV_* values derived from measured spICP-MS and HR-SEM size distributions and *PNC_direct_* values from spICP-MS for a set of 14 gold nanoparticle suspensions of varying particle sizes and surface coatings. Reassuringly, no consistent method-correlated biases were seen. However, inconsistent variability between methods for the different individual suspensions was observed, pointing to unknown and unstable sources of bias that limit confidence in both the derived and direct PNC measurements to about ±5% for any specific suspension within this set of samples. Continued research into the sources of bias and general lack of consistency for PNC measurement methods is needed if lower uncertainty is to be reliably obtained.

A limitation in calculating *PNC_AMV_* is that it requires accurate knowledge of the complete particle size distribution, which is often not available. We have derived an equation that exactly corrects *PNC_AMD_* to *PNC_AMV_* for normal distributions with known AMD and CV. In addition, since the skew dependence of the *PNC_AMD_* error is small, the general use of this same CV-only based correction factor, even when the shape of the particle distribution is unknown, will typically correct *PNC_AMD_* to within 3% of *PNC_AMV_* for most monomodal distributions with CV less than 0.35. This covers the great majority of nanoparticle suspensions encountered in our experience. We therefore strongly advocate for the general use of this correction to *PNC_AMD_* whenever the complete particle size distribution is not available.

The derivation described in this paper using AMV to calculate PNC has the potential to increase the quality of PNC measurements wherever they are used in nanotechnology applications by correcting a key bias frequently encountered in current practice. Accurate determination of the PNC is of critical importance in many contexts such as nanomedicine dosing or nanomaterial and nanoplastic contamination and toxicity testing. Application of the *PNC_AMV_* also offers a new quality control metric for spICP-MS measurements; if *PNC_direct_* and *PNC_AMV_* values are not in agreement, an unknown source of bias is implied that should be investigated. Furthermore, this comparison could provide an indicator of the technical proficiency for each participant in an interlaboratory comparison. Progress in reducing the difference between *PNC_direct_* and *PNC_AMV_* will help improve concordance in interlaboratory comparisons and support the development of low-uncertainty PNC-certified reference materials.

## Abbreviations

AMD: arithmetic mean diameter
AMV: arithmetic mean volume
bPEI: branched polyethylenimine
CV: coefficient of variation
HMV: harmonic mean volume
HR-SEM: high-resolution scanning electron microscopy
PDF: probability density function
PEG: polyethylene glycol
PNC: particle number concentration
PVP: polyvinylpyrrolidone
RM: reference material
spICP-MS: single-particle inductively coupled plasma mass spectroscopy
SRM: standard reference material
TMC: total mass concentration

## Variables

*C*: total mass concentration of the particle material
*CV*: coefficient of variation of distribution (σ/μ)
γ_1_: skewness of distribution (aka standardized third moment) *m*_3_/*m*_2_^3/2^,where *m*_3_ = (1/*N*) ∑(*x*_*i*_–*x̅*)^3^ and *m*_2_ = (1/*N*) ∑(*x*_*i*_–*x̅*)^2^
κ: correction factor for the AMD-derived PNC
μ: arithmetic mean of distribution
*N*: total number of particles
*PNC*: particle number concentration variable
*PNC_AMD_*: PNC calculated from *C,* using $V_{\mathrm{s̅}}$ as the particle volume
*PNC_AMV_*: PNC calculated from *C,* using ${\mathrm{V̅}}_{A}$ as the particle volume
*PNC_avg_*: average of *PNC_direct_* and *PNC_AMD_* from both HR-SEM and spICP-MS
*PNC_direct_*: PNC derived from counting events in spICP-MS
*PNC_HMC_*: PNC calculated as a harmonic mean of *PNC*_*i*_
*PNC_HMV_*: PNC calculated from *C,* using ${\mathrm{V̅}}_{H}$ as the particle volume
*PNC_*i*_*: PNC calculated from *i*th particle size
ρ: particle density
*s*_i_: spherical-equivalent-volume diameter of the *i*th particle
*s̅*: arithmetic mean diameter of particles. $\left(\frac{1}{N}\sum s_{i}\right)$
σ: standard deviation
*V*: particle volume
*V*_*i*_: volume of the *i*th particle
${\mathrm{V̅}}_{A}$: arithmetic mean volume of the particles, ( $\frac{1}{N}\sum V_{i}$ )
${\mathrm{V̅}}_{H}$: harmonic mean volume of the particles, ${\left[\frac{1}{N}\sum \frac{1}{V_{i}}\right]}^{-1}$
$V_{\mathrm{s̅}}$: volume of a particle having arithmetic-mean-diameter size, [ $\frac{\pi }{6}{\left(\mathrm{s̅}\right)}^{3}$ ]
